# Efficacy of first-line tyrosine kinase inhibitor between unresectable stage III and stage IV EGFR-mutated non-small cell lung cancer patients

**DOI:** 10.18632/aging.204781

**Published:** 2023-06-08

**Authors:** Yang Liu, Huan-Wei Liang, Xin-Bin Pan

**Affiliations:** 1 Department of Radiation Oncology, Guangxi Medical University Cancer Hospital, Nanning 530021, Guangxi, P.R. China

**Keywords:** non-small cell lung cancer, NSCLC, epidermal growth factor receptor, EGFR, tyrosine kinase inhibitor

## Abstract

Purpose: To compare survivals between unresectable stage III and stage IV EGFR-mutated non-small cell lung cancer (NSCLC) patients receiving first-line EGFR-TKI.

Materials and methods: Unresectable stage III and stage IV EGFR-mutated NSCLC patients were investigated from September 2012 to May 2022. Patients received EGFR-TKI as the first-line treatment. Progression-free survival (PFS) and overall survival (OS) were assessed using the Kaplan-Meier method and propensity score matching (PSM) analyses.

Results: A total of 558 patients were included: 478 (85.66%) patients were stage IV and 80 (14.34%) patients were stage III. Before PSM, stage III patients showed a better median PFS (15 vs. 13 months; *P*=0.026) and a similar median OS (29 vs. 30 months; *P*=0.820) compared to stage IV patients. Stage IV was an independent prognostic factor for PFS [hazard ratio (HR)=1.47, 95% confidence interval (CI): 1.06-2.04; *P*=0.021], but not for OS (HR=1.11, 95% CI: 0.77-1.60; *P*=0.560). After PSM, a better median PFS (15 vs. 12 months; *P*=0.016) and a similar median OS (29 vs. 30 months; *P*=0.960) were found between stage III and stage IV patients.

Conclusions: OS was similar between unresectable stage III and stage IV EGFR-mutated NSCLC patients receiving EGFR-TKI as the first-line treatment.

## INTRODUCTION

Concurrent chemoradiotherapy is recommended for unresectable stage III non-small cell lung cancer (NSCLC) patients [1–6]. Epidermal growth factor receptor (EGFR) wild-type cases are further advised to receive durvalumab [7–10]. For patients with EGFR mutation, immunotherapy is not recommended. Concurrent chemoradiotherapy is still the optimal treatment for these patients. However, several studies suggested that concurrent chemoradiotherapy might lead to worse survivals in EGFR-mutated patients compared with those of EGFR wild-type patients [11, 12].

On the other hand, several clinical trials have proved that EGFR-tyrosine kinase inhibitor (TKI) is the standard treatment for stage IV EGFR-mutated patients [13–19]. In these trials, a part of included patients were stage III diseases. However, survivals between stage IV patients and stage III patients receiving EGFR-TKI have not been assessed. We aimed to investigate survivals between stage IV and stage III EGFR-mutated NSCLC diseases receiving first-line EGFR-TKI.

## RESULTS

### Patient characteristics

This study included 558 patients: 478 (85.66%) patients were stage IV and 80 (14.34%) patients were stage III. Table 1 shows the patient characteristics before and after PSM. Before PSM, clinical factors, including ECOG performance status, smoking status, T stages, N stages, EGFR subtypes, and treatment patterns were not balanced. After PSM, 74 stage IV cases and 74 stage III cases were matched. All clinical factors were balanced after PSM (*P*>0.05). For stage III cases, the median follow-up time was 19 [interquartile range (IQR): 11-30] months. For stage IV cases, the median follow-up time was 20 (IQR: 12-31) months. Twenty-six patients were lost to follow-up. The follow-up rate was 95.34%.

**Table 1:** Patient characteristics.

	**The unmatched cohort**		***P***	**The PSM cohort**		***P***
	**Stage III (n=88)**	**Stage IV (n=408)**		**Stage III (n=88)**	**Stage IV (n=408)**	
Age			0.060			0.999
≤59	32 (40.0%)	249 (52.1%)		32 (43.2%)	31 (41.9%)	
>59	48 (60.0%)	229 (47.9%)		42 (56.8%)	43 (58.1%)	
Sex			0.875			0.185
Female	41 (51.2%)	253 (52.9%)		37 (50.0%)	46 (62.2%)	
Male	39 (48.8%)	225 (47.1%)		37 (50.0%)	28 (37.8%)	
ECOG			<0.001			0.742
0	43 (53.8%)	162 (33.9%)		38 (51.4%)	35 (47.3%)	
1	37 (46.2%)	227 (47.5%)		36 (48.6%)	38 (51.4%)	
2	0 (0.0%)	75 (15.7%)		0 (0.0%)	1 (1.3%)	
3	0 (0.0%)	14 (2.9%)				
Smoking status			0.009			0.138
Never smoker	59 (73.8%)	363 (75.9%)		54 (73.0%)	60 (81.1%)	
Former smoker	17 (21.2%)	113 (23.6%)		16 (21.6%)	14 (18.9%)	
Current smoker	4 (5.00%)	2 (0.42%)		4 (5.4%)	0 (0.0%)	
T stage			0.001			0.635
T1	18 (22.5%)	71 (14.9%)		14 (18.9%)	18 (24.3%)	
T2	29 (36.3%)	123 (25.7%)		27 (36.5%)	28 (37.8%)	
T3	13 (16.2%)	56 (11.7%)		13 (17.6%)	8 (10.8%)	
T4	20 (25.0%)	194 (40.6%)		20 (27.0%)	20 (27.0%)	
unknown	0 (0.0%)	34 (7.1%)				
N stage			0.002			0.742
N0	2 (2.5%)	46 (9.6%)		2 (2.7%)	2 (2.7%)	
N1	2 (2.5%)	28 (5.9%)		2 (2.7%)	5 (6.8%)	
N2	31 (38.8%)	154 (32.2%)		30 (40.5%)	28 (37.8%)	
N3	45 (56.2%)	215 (45.0%)		40 (54.1%)	39 (52.7%)	
unknown	0 (0.0%)	35 (7.3%)				
EGFR			0.008			0.276
Exon 19 deletion	45 (56.3%)	241 (50.4%)		40 (54.1%)	47 (63.5%)	
L858R mutation	29 (36.2%)	131 (27.4%)		28 (37.8%)	19 (25.7%)	
Other	6 (7.5%)	106 (22.2%)		6 (8.1%)	8 (10.8%)	
Treatments			0.009			0.182
TKI	55 (68.8%)	309 (64.7%)		51 (68.9%)	54 (73.0%)	
TKI+chemotherapy	23 (28.7%)	101 (21.1%)		21 (28.4%)	14 (18.9%)	
TKI+antiangiogenic therapy	2 (2.50%)	68 (14.2%)		2 (2.7%)	6 (8.1%)	

PSM, propensity score matching; ECOG, Eastern Cooperative Oncology Group; EGFR, epidermal growth factor receptor; TKI, tyrosine kinase inhibitor.

### Treatment patterns

**Figure 1:**
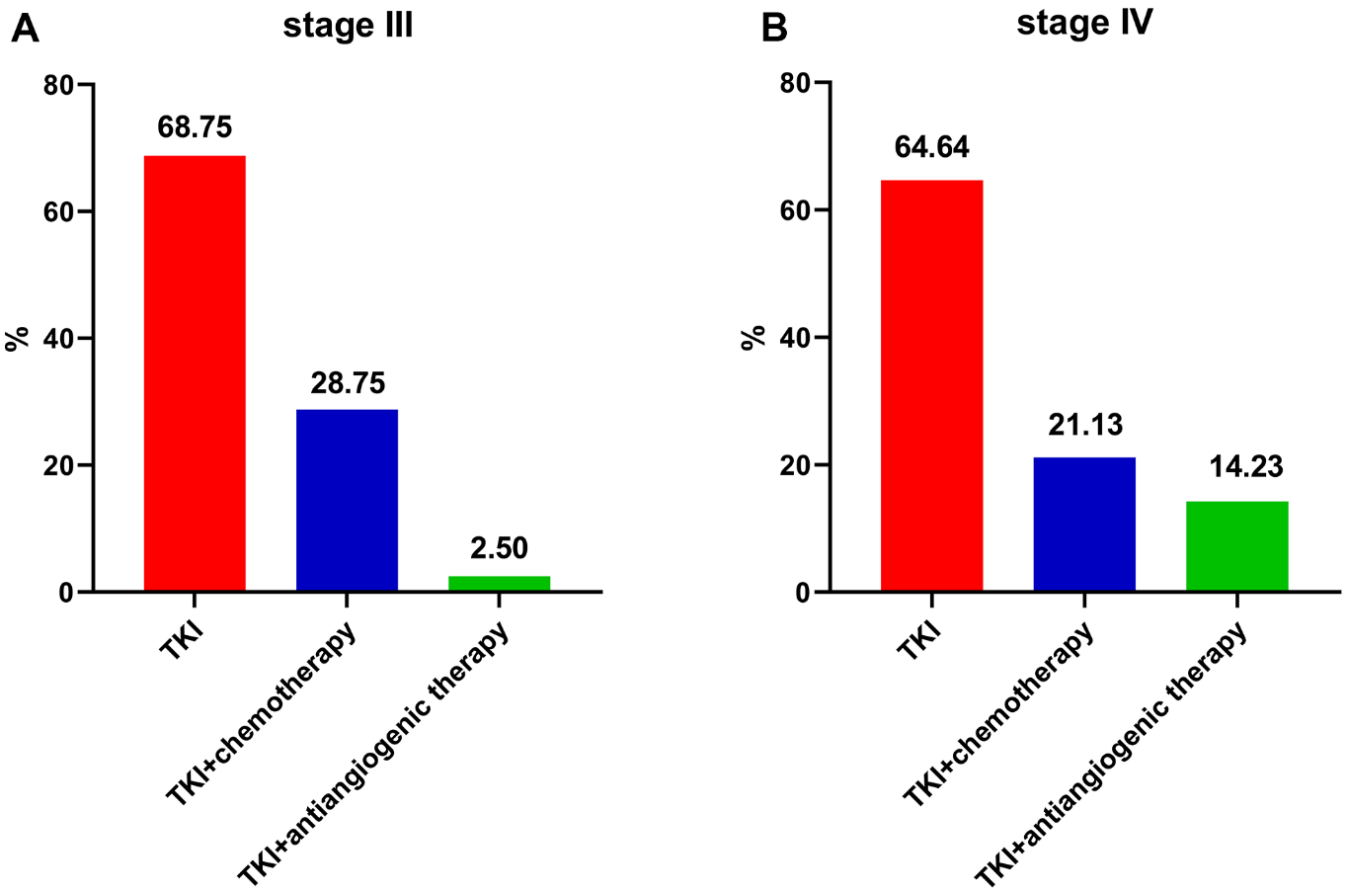
**Frequency of initial treatment modalities for EGFR-mutated non-small cell lung cancer patients.** (**A**) stage III. (**B**) stage IV. EGFR: Epidermal growth factor receptor. TKI: tyrosine kinase inhibitor.

Initial therapy included 3 treatment regimens including TKI therapy, TKI plus chemotherapy, and TKI plus antiangiogenic therapy. Figure 1 shows the initial treatment patterns. Among stage III patients, 68.75%, 28.75%, and 2.50% patients received TKI, TKI plus chemotherapy, and TKI plus antiangiogenic therapy, respectively. Among stage IV patients, 64.64%, 21.13%, and 14.23% patients received TKI, TKI plus chemotherapy, and TKI plus antiangiogenic therapy, respectively.

### Survivals before PSM

**Figure 2:**
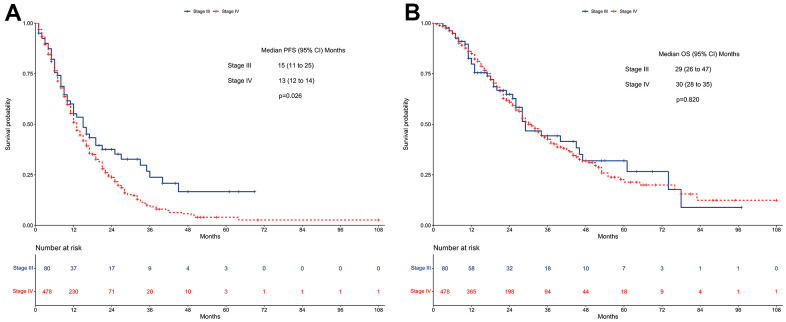
**Survivals between stage III and stage IV EGFR-mutated non-small cell lung cancer patients receiving TKI therapy in the unmatched cohort.** (**A**) Progression-free survival. (**B**) Overall survival. EGFR: Epidermal growth factor receptor. TKI: tyrosine kinase inhibitor.

Stage III patients showed a better median PFS than stage IV patients (15 vs. 13 months; *P*=0.026, Figure 2A). In contrast, the median OS did not differ between stage III cases and stage IV cases (29 vs. 30 months; *P*=0.820, Figure 2B).

**Figure 3:**
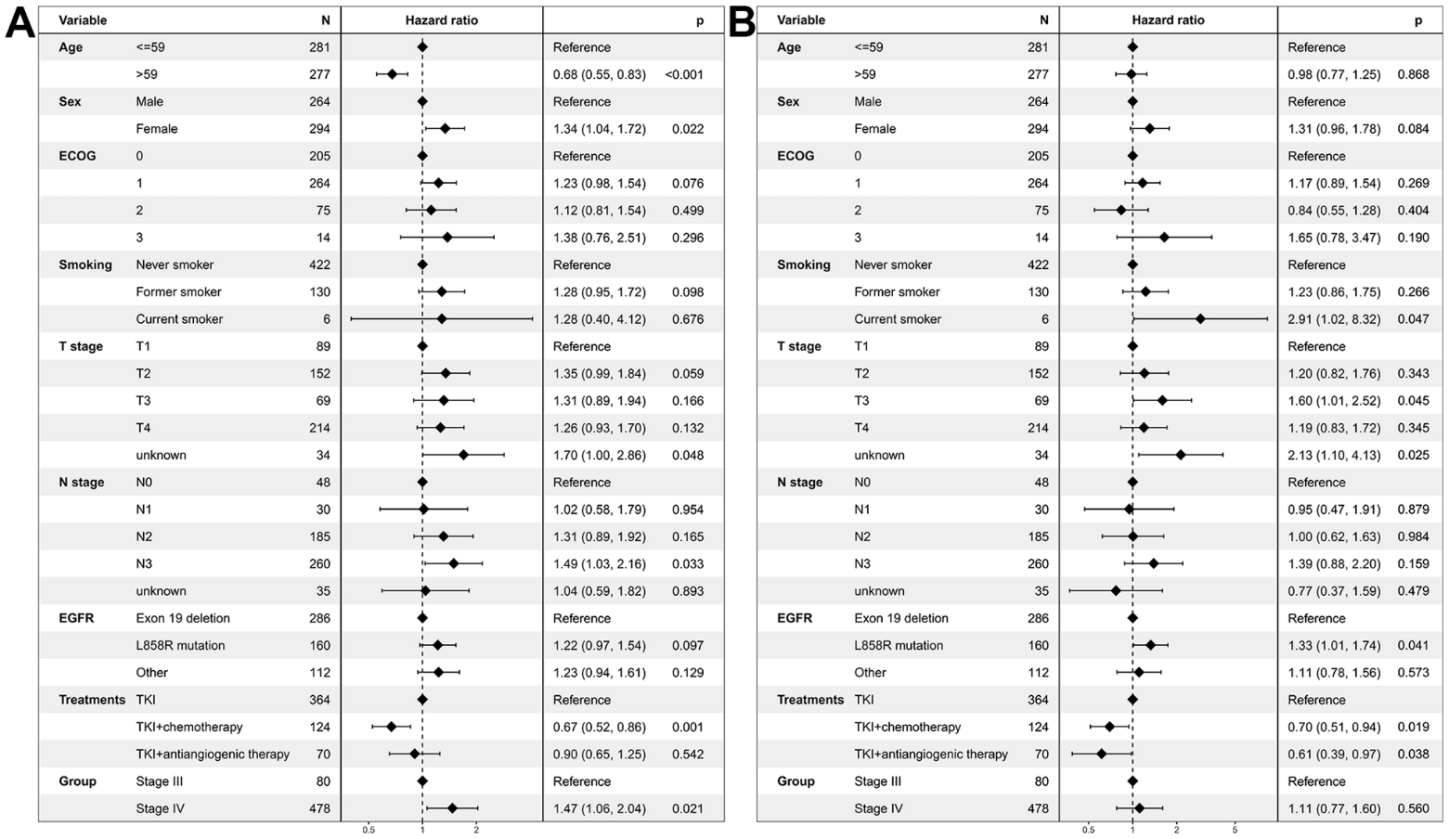
**Multivariate regression analysis of prognostic factors for EGFR-mutated non-small cell lung cancer patients receiving TKI therapy in the unmatched cohort.** (**A**) Progression-free survival. (**B**) Overall survival. EGFR: Epidermal growth factor receptor. TKI: tyrosine kinase inhibitor. ECOG: Eastern Cooperative Oncology Group.

Multivariate regression analysis revealed that stage IV patients had a worse PFS compared with stage III patients (HR=1.47, 95% CI: 1.06-2.04; *P*=0.021, Figure 3A). In contrast, no difference in OS was found between stage IV patients and stage III patients (HR=1.11, 95% CI: 0.77-1.60; *P*=0.560, Figure 3B).

### Survivals after PSM

**Figure 4:**
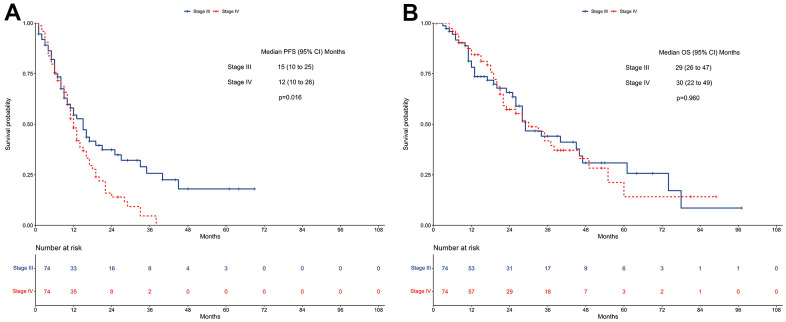
**Survivals between stage III and stage IV EGFR-mutated non-small cell lung cancer patients receiving TKI therapy in the propensity-matched cohort.** (**A**) Progression-free survival. (**B**) Overall survival. EGFR: Epidermal growth factor receptor. TKI: tyrosine kinase inhibitor.

Stage III patients had a better median PFS than stage IV patients (15 vs. 12 months; *P*=0.016, Figure 4A). In contrast, the median OS did not differ between stage III cases and stage IV cases (29 vs. 30 months; *P*=0.960, Figure 4B).

**Figure 5:**
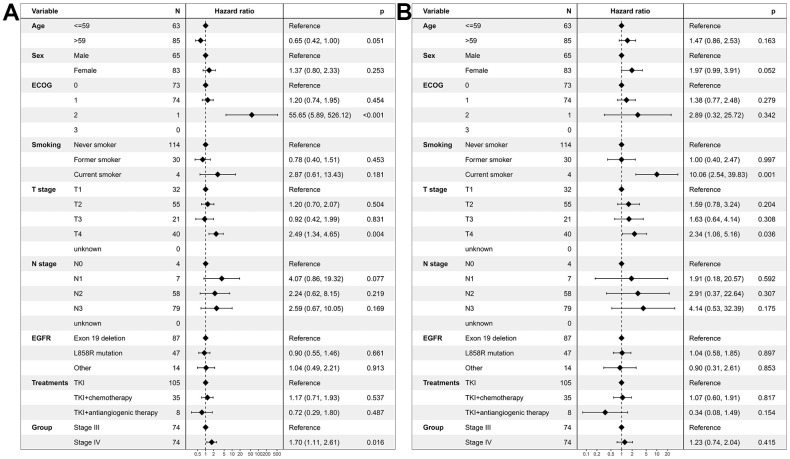
**Multivariate regression analysis of prognostic factors for EGFR-mutated non-small cell lung cancer patients receiving TKI therapy in the propensity-matched cohort.** (**A**) Progression-free survival. (**B**) Overall survival. EGFR: Epidermal growth factor receptor. TKI: tyrosine kinase inhibitor. ECOG: Eastern Cooperative Oncology Group.

Multivariate regression analysis revealed that stage IV patients had a worse PFS compared with stage III patients (HR=1.70, 95% CI: 1.11-2.61; *P*=0.016, Figure 5A). In contrast, no difference in OS was found between stage IV patients and stage III patients (HR=1.23, 95% CI: 0.74-2.04; *P*=0.415, Figure 5B).

## DISCUSSION

This retrospective study suggested that stage III patients receiving first-line EGFR-TKI had a better PFS compared with stage IV patients. However, improved PFS did not translate into the benefit of OS. The OS between stage III patients and IV patients were not different.

It was reported that unresectable EGFR-mutated stage III patients were associated with worse PFS treated with concurrent chemoradiotherapy compared with EGFR wild type [11, 12, 20]. The median PFS ranged from 6.3 to 8.9 months. In our study, stage III patients receiving first-line EGFR-TKI revealed a median PFS of 15 months. The similar results were reported by previous studies [21–24]. These results were better than that of concurrent chemoradiotherapy. According to these findings, most unresectable stage III patients were treated with first-line EGFR-TKI treatment instead of concurrent chemoradiotherapy.

However, our study suggested that the median OS between stage III cases and stage IV cases was comparable. This result might be caused by a fact that stage III patients included in our study did not receive any local therapy (radiotherapy or surgery). It was reported that first-line EGFR-TKI treatment alone had poor prognosis in OS (HR=1.983, 95% CI: 1.079-3.643; *P*=0.0273) for stage III patients [20]. Patients receiving first-line EGFR-TKI treatment alone showed a median OS of 25.4 months, which was similar to our study [20].

Reasons of no local treatments in stage III patients might be the following factors. First, locally directed concurrent chemoradiotherapy is given with curative intent for stage III patients. Adjuvant TKI treatment after concurrent chemoradiotherapy might provide potential benefits [20, 22]. However, clinical guidelines are not well followed in stage III patients in clinical practice. Second, lack of radiation department and surgery departments in defining the treatment approaches. Even in patients who suffered from treatment failures after first-line treatments, local treatments (radiotherapy or surgery) were missed.

There were some limitations in this study. First, a total of 80 (14.34%) stage III patients were included. It might be not sufficient for statistical analysis comparing the survivals between stage IV patients and stage III patients. The statistical power of the analysis might be reduced. Second, the present study was a retrospective cohort study. Selection biases existed in this study. We performed several analytic methods, including multivariate adjustment and PSM, to control potential biases. Both cox proportional hazard regression and PSM revealed a consistent result that stage III patients showed a better PFS and a similar OS compared with stage IV patients.

In conclusion, the current study revealed that no statistically significant difference in OS was observed between stage III and IV EGFR-mutated NSCLC patients receiving EGFR-TKI as the first-line treatment.

## MATERIALS AND METHODS

### Patients

NSCLC cases were searched in Guangxi Medical University Cancer Hospital from September 2012 to May 2022. Inclusion criteria: (1) lung adenocarcinoma, (2) EGFR mutation, (3) stage IV and III for the 8^th^ edition American Joint Committee on Cancer staging system. Exclusion criteria: (1) incomplete data, (1) adenosquamous carcinoma, (3) EGFR subtypes unknown, (4) patients did not receive any treatments, (5) patients included in clinical trials, (6) patients received surgery, (7) patients received radical radiotherapy.

Clinical factors, including age, sex, Eastern Cooperative Oncology Group (ECOG) performance status, smoking status, T stages, N stages, AJCC stages, EGFR subtypes, and treatment patterns (TKI therapy, TKI plus chemotherapy, and TKI plus antiangiogenic therapy) were extracted.

### Endpoints

Treatment failures were determined according to pathology reports and/or imaging reports. Death was determined from the statements. Progression-free survival (PFS) was the primary endpoint, which defined as the duration from the date of diagnosis to the date of progression or death. Overall survival (OS) was the secondary endpoint, which defined as the duration from the date of diagnosis to the date of death.

### Statistical analysis

According to the median value, the continuous factor of age was transformed to categorical factor. Categorical factors, including age, sex, ECOG performance status, smoking status, T stages, N stages, EGFR subtypes, and treatment patterns were compared between stage IV and stage III groups using the χ^2^ test or Fisher’s exact test.

Kaplan-Meier analysis with log-rank test statistics was used to compare median PFS and OS between stage IV and stage III groups. Cox proportional hazards models adjusted for age, sex, smoking status, ECOG performance status, T stages, N stages, treatment patterns, EGFR subtypes, and clinical stages were used to estimate the hazard ratios (HRs) with 95% confidence intervals (CIs) for potential independent prognostic factors.

This study performed a matched case-control analysis to reduce the influence of selection bias on the comparison of outcomes between stage IV and stage III patients using propensity score matching (PSM). In the process of calculating the propensity scores, stage III patients were taken as the dependent variable. One-to-one matching without replacement was completed in a logistic regression model. The nearest-neighbor match of the propensity score for factors (age, sex, smoking status, ECOG performance status, T stages, N stages, treatments, and EGFR subtypes) was 0.05 caliper on the logistic regression model.

This study used R software (version 4.2.1) and SPSS Statistics Version 26.0 software (IBM Co., Armonk, NY, USA) to perform statistical analyses. All tests were two-sided. *P*<0.05 was considered statistically significant.
